# Prevalence of Restless Legs Syndrome among Medical Students of Karachi: An Experience from a Developing Country

**DOI:** 10.1155/2020/7302828

**Published:** 2020-02-18

**Authors:** M. Ishaq, S. U. Riaz, N. Iqbal, S. Siddiqui, A. Moin, S. Sajjad, T. Ali, S. Jamali

**Affiliations:** Jinnah Medical & Dental College, Pakistan

## Abstract

**Background:**

Restless legs syndrome (RLS) is a neurological disorder characterized by an uncomfortable sensation in the legs which gets worse in the evening or night, relieved upon movement. The aim of this study was to specify the prevalence of RLS in the group of young medical students and to assess the effect of RLS on sleep, as sleep disturbance is one of the chief complaints of RLS patients. We also studied its association with smoking as it is considered an aggravating factor.

**Method:**

This was a cross-sectional study conducted from June 2017 to July 2018 in Karachi. A total of 300 students (220 females and 80 males) participated and were given questionnaires to detect RLS based on criteria proposed by the International Restless Legs Syndrome Study Group. Subjects who were positive for RLS were further asked questions about sleep by using the Epworth Sleepiness Scale and severity of RLS by using RLS Rating Scale. They were also asked about their smoking status.

**Results:**

The frequency of RLS is 8% among young adults. Out of 300 medical students, 24 students were classified positive for RLS with a female preponderance (66.7% were females and 33.3% were males). The severity of RLS was more rated to be mild to moderate. The effect of RLS on sleep was in the mild range. The *p* value of smoking status comparing with gender came out to be <0.001, and *p* value of smoking status comparing with gender came out to be <0.001, and

**Conclusion:**

It is concluded that we found RLS to be present significantly in our population that is without comorbidities. Our results showed female preponderance and a mild sleep disturbance in our study population. More attention is needed to recognize RLS and to manage the aggravating factors of RLS.

## 1. Introduction

Restless legs syndrome (RLS) is a neurological disease characterized by throbbing, pulling, creeping, or other unpleasant sensations in the legs and an uncontrollable and sometimes overwhelming urge to move them [1]. It was first described by Sir Thomas Willis, an English physician in 1672 [2]. Later in 1945, Ekbom classified it into a sensory form called as asthenia crurum paraesthetica and a painful variant called asthenia crurum dolorosa [3].

The most distinctive aspect of the condition is that lying down and trying to relax activates these symptoms. Most people with RLS have difficulty falling asleep or staying asleep. Left untreated, the condition causes exhaustion and daytime fatigue. Many people with RLS report that their job, personal relations, and activities of daily living are strongly affected as a result of their sleep deprivation. They are often unable to concentrate, have impaired memory, or fail to accomplish daily tasks. It also can make traveling difficult and can cause depression [1]. For 60-70% of patients with RLS diagnosis, sleep disturbance is the chief cause leading them to seek treatment [4–6]. RLS has been associated with dopaminergic dysregulation, several health conditions, and various lifestyle factors like consumption of alcohol, coffee, cigarettes, and some drugs [7].

RLS has also been found to substantially increase the risk of major depressive disorder and anxiety disorders [8]. Restless legs syndrome is usually diagnosed according to the criteria proposed by International RLS Study Group (IRLSSG) [4]. International Restless Legs Syndrome Study Group rating scale (IRLSS) is a valid instrument that has shown high internal consistency, test-retest reliability, and clinical validity [9]. This is primarily a neurobehavioral disorder; hence, objectively, severity can only be assessed by using a questionnaire. Restless legs syndrome affects the quality of sleep and causes daytime somnolence; this was calculated by the Epworth Sleepiness Scale [10].

RLS is one of the underdiagnosed conditions globally as well as in Pakistan, and there is scarce local data related to the prevalence of RLS among the young population. The purpose of our study is to find out the prevalence and to assess the severity of symptoms among medical students. Also, its effect on sleep of students will be studied. This will help create awareness about RLS among medical students. Also, any substantial issues found through our study will help lessen the burden of this disease and implement the strategies necessary for treatment to bring about change.

## 2. Methodology

### 2.1. Study Design

It was a cross-sectional observational study.

### 2.2. Study Setting

This study was conducted in different medical colleges of Karachi, Pakistan, that included Jinnah Medical and Dental College (JMDC), Karachi Medical and Dental College (KMDC), Dow International Medical College (DIMC), Liaquat College of Medicine and Dentistry (LCMD), and Al-Tibri Medical College (ATMC).

### 2.3. Inclusion and Exclusion Criteria

Our study population consisted of MBBS undergraduate students only. Individuals aged 18-26 were included. Individuals who had impaired sensation, neuropathies, pregnancy, morbid obesity, decreased vitamin B 12, vegetarians, anemic, thyroid issues, known comorbidities, history of trauma to the limbs, and any febrile illnesses and pregnancy proven by biochemical evidence were excluded. The study was approved by the ethical committee of Jinnah Medical and Dental College, and individual consent was obtained through verbal and implied consent on the questionnaire.

### 2.4. Sampling Technique and Sample Size

The subjects were selected through nonprobability convenience sampling.

The sample size of the study was calculated by using the formula:

(1) $$\begin{array}{c} n=\frac{{\left(Z\right)}^{2}P\left(1‐P\right)}{d^{2}}, \end{array}$$

where *n* is the sample size, *Z* is the *Z* statistic for a level of confidence, *P* is the expected prevalence or proportion (if the expected prevalence is 20%, then *P* = 0.2), and *d* is the precision (If the precision is 5%, then *d* = 0.05).

Assuming a prevalence of 23.6% (16), keeping the confidence level at 95% and accepting a 5% margin of error, the estimated sample size was calculated to be 278. Further adding 10% for nonresponse, missing values, and dropouts, we needed 300 subjects. For ease and diversity, we visited 4 different medical institutions and included the sample size subjects.

### 2.5. Study Tool and Data Collection Method

Data was collected from June 2017 to June 2018. After evaluating the patients' eligibility to participate in the study and obtaining consent, study questionnaires, which were validated and were in the English language (all participants being medical students know it), were then administered to the subjects, and they were interviewed to rule out other conditions that mimic RLS.

The study questionnaire was to be divided into four parts. The first part of the questionnaire was to gather demographic information including age, gender, and smoking status of the subject. The second part was to inquire about the essential questions of the criteria of RLS specified by IRLSSG [4]:

1. An urge to move the legs, usually accompanied or caused by uncomfortable and unpleasant sensations in the legs. (Sometimes the urge to move is present without the uncomfortable sensations, and sometimes the arms or other body parts are involved in addition to the legs.)
2. The urge to move or unpleasant sensations begin or worsen during periods of rest or inactivity such as lying or sitting
3. The urge to move or unpleasant sensations are partially or totally relieved by movement, such as walking or stretching, at least as long as the activity continues
4. The urge to move or unpleasant sensations are worse in the evening or night than during the day or only occur in the evening or night. (When symptoms are very severe, the worsening at night may not be noticeable but must have been previously present.)

If the answer to the questionnaire questions was yes and there is no known cause apart from RLS being responsible for these symptoms, so this was definitive for RLS. The third and fourth parts of the questionnaire relating to severity and sleepiness were only administered to the RLS-positive individuals. In the third part, questions regarding sleepiness were asked using Epworth Sleepiness Scale [10]. This scale is important in clinics to measure the improvement with treatment as well as for the research purpose to measure the severity of RLS. To know about its impact on sleep, the Epworth Sleepiness Scale was used. This scale intended to measure daytime sleepiness and is helpful in the diagnosis of sleep disturbance among medical students.

In the fourth part, the severity of RLS symptoms was explored by using the RLS Rating Scale. Different questions regarding RLS symptoms were asked from the subjects using this scale, and score was recorded. According to the RLS Severity Rating Scale, a score of 1-10 denoted mild severity, 11-20 was moderate, 21-30 was severe, and 31-40 was very severe. Statistical analysis was performed using the program Statistical Package for the Social Sciences 22.0 (SPSS). The qualitative variables were expressed as percentages, and quantitative variables were expressed as mean ± standard deviation. A value < 0.05 was regarded as statistically significant.

## 3. Results

Around 390 students were approached in medical colleges, and the data of 300 subjects were included in the final analysis. The data was collected from different medical colleges (LCMD, JMDC, DIMC, KMDC, and Al-Tibri) of Karachi. Overall, there were 220 (73.3%) females and 80 (26.6%) males. Out of 300 students, 33 (11%) were smokers and 267 (89%) were nonsmokers. The mean age was calculated to be 21.3 (SD = 1.68). Demographic characteristics of the sample population are summarized in Table 1.

**Figure 1:**
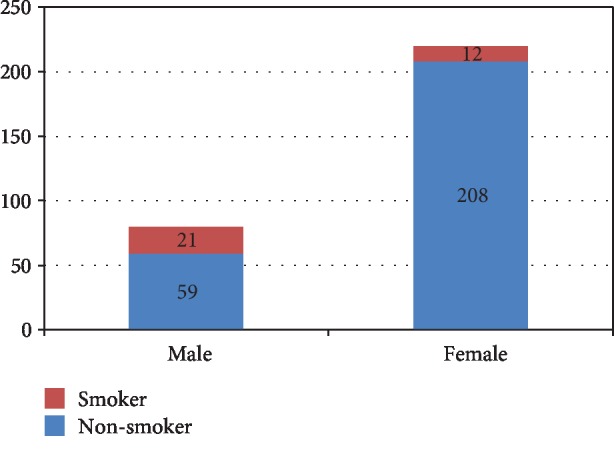
Bar graph showing the frequency of smokers and nonsmokers among male and female students.

Out of 80 male students, 21 (26.25%) were smokers and the rest were nonsmokers. In female students, 12 (5.45%) out of 220 were smokers and 208 (94.54%) were nonsmokers (Figure 1).

**Figure 2:**
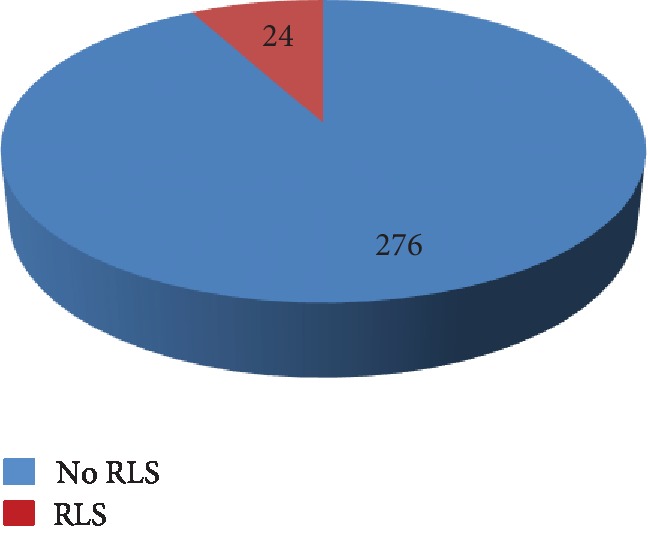
Frequency of RLS among medical students.

Out of 300 individuals, 24 (8%) were classified as RLS positive based on criteria defined by IRLSSG shown in Figure 2. The prevalence of RLS was found to be 8.6% and 9.3% among females and males, respectively.

Out of 300 students, 21 (7%) males were smokers and 59 (19.6%) were nonsmokers and 12 (4%) females were smokers and 208 (69.33) were nonsmoker. In those smoker males, 7 were RLS positive and 73 were negative whereas 17 smoker females were found to be RLS positive and 203 were negative, respectively. The *p* value of smoking status comparing with gender came out to be <0.001, and *p* value of RLS is 0.773 in Table 2.

The mean (SD) value of RLS in males is 11.57 (5.798) compared with the mean (SD) value of RLS females which is 10.86 (4.384). The mean (SD) value of severity of sleepiness of RLS in males is 9.71 (3.039) as compared to the severity of sleepiness of RLS in females which is 6.866 (3.020). The *p* value calculated was 0.754 comparing between gender and RLS severity score mean. The *p* value was 0.053 comparing between gender and sleepiness severity score in Table 3.

The students diagnosed with RLS were 24, out of which only 21 solved the questionnaire part for assessing severity based upon the RLS Rating Scale. Out of those 21 students, 9 (42.85%) students had mild and 12 (57.14%) students had moderate severity of RLS symptoms.

Out of them as shown in Table 4, 3% were males and 6% were females who had mild RLS severity and 4% males and 8% females had moderate RLS severity. In Sleepiness severity scale, 6 females reported no daytime sleepiness. Among students, 4 males and 8 females reported mild severity. Only 1 male and 1 female had moderate sleepiness severity. Two males had severe daytime sleepiness. There were no males or females with very severe sleep symptoms.

## 4. Discussion

We went through various research articles, and to the best of our knowledge, this was the first attempt to categorize RLS among the young generation in Pakistan in an attempt to find out its repercussions and aftermath on sleeping habits of students who suffer from this neurological disorder. Since pain in legs is a common complaint in all age groups, the data in our population supporting is scarce. As far as we know, this is the only initiative taken among medical students with respect to this topic in Pakistan.

Mahmood et al. made attempts to see the prevalence of RLS in the general population of Karachi. Their results, although much higher than ours at 8% compared to theirs at 23.6%, confirmed female preponderance. There was no specified age group defined, and they took patients with comorbidities. Our study was in healthy individuals, so the impact is foreseen to be higher with advanced age and comorbidities [11]. Both studies go on to prove a ratio of 2 : 1 of female to male suffering from this condition. A similar study was carried out among medical students in Egypt showing a prevalence of 11.8% with a female preponderance. Most had idiopathic variety [12, 13].

Our study reemphasizes the fact that RLS is more of a disease in the female gender than in the male gender, a point highlighted by the previous studies. We have four such studies to confirm our conclusions [4, 5, 14, 15]. Since our sample was of a university-going age group, the severity of the symptoms was of mild category. It also can be because the students were suffering from no comorbidities and were young and healthy [16, 17]. All these studies concluded, much like ours, that patients suffering from RLS were subject to some or to the other sort of sleep disturbance. [5, 15, 18, 19].

A study published in *Sleep Medicine* in 2015 stated that students self-diagnosed themselves with restless legs syndrome and were not aware of the term Willis-Ekbom Disease [20]. A study with a higher subject participation was conducted in Iran where patients with all sort of comorbid factors were investigated, and it concluded that smoking or any neurodegenerative comorbidity played a significant role in the incidence [21]. A study in Turkey showed a general low incidence, but people living at high altitudes were at a greater risk to develop this disorder, so more studies can be done to probe into this topic in the future. This study also went on to reiterate the role that smoking plays in acquiring RLS [22].

Various meta-analysis has been stating the causes of restless legs syndrome and showed iron deficiency, uremia, diabetes, and certain drugs play a role in increased incidence [23, 24]. One of the strengths of this study is that diagnosis was made according to the criteria set by IRLSSG for diagnosing RLS.

## 5. Conclusion

It has been concluded that the prevalence of RLS among the young population of Karachi cannot be underestimated. It is also an underdiagnosed condition as most people did not know the condition responsible for their symptoms in our research.

### 5.1. Limitations

One lack of our study was that the sample size was too small for whom severity and effect on sleep were studied. Nevertheless, for assessing severity and effect on sleep, we had used internationally established scales which had test-retest ability. This study cannot be generalized as it was done in young and healthy subjects, and there is a need for further probing into the topic keeping in mind the population with comorbidities. We also noted that the diagnostic questions asked in the questionnaires administered to the students were answered based on self-judgment instead of a complete clinical interview and examination.

### 5.2. Recommendations

Patients with RLS should be advised to see the physician for early recognition and treatment, and further research is warranted to improve and recognize the condition in patients of our population. Therefore, awareness needs to be spread about RLS. As it also affects the sleep of students, thus, more extensive studies are required to be done about the etiologies behind these symptoms so that they can be addressed then.

## Figures and Tables

**Table 1:** Baseline characteristics of study respondents (*n* = 300).

Variables	Mean (SD)	*n* (%)
Age (year)		21.3 (1.68)
Gender		
Male		80 (26.6)
Female		220 (73.3)
Smoker		
Yes		267 (89)
No		33 (11)

SD: standard deviation.

**Table 2:** Association of gender with smoking and RLS (*n* = 300).

Variable	Females *n* (%)	Males *n* (%)	*p* value
Smoking status			
Smoker	12 (36.4)	21 (63.6)	<0.001
Nonsmoker	208 (77.9)	59 (22.1)	
RLS			
Yes	17 (70.8)	7 (29.2)	0.773
No	203 (73.6)	73 (26.4)	

*p* value calculated by using the chi-squared test.

**Table 3:** Comparison of RLS rating score and sleepiness severity score among gender (*n* = 21).

Variable	Females Mean (SD)	Males Mean (SD)	*p* value^a^
RLS score (rating score)	10.86 (4.348)	11.57 (5.798)	0.754
Sleepiness severity score	6.866 (3.020)	9.71 (3.039)	0.053

^a^ *p* value calculated by using an independent sample *t*-test.

**Table 4:** Distribution of RLS and sleepiness scale severity among gender.

Variable	Females *n* (%)	Males *n* (%)
RLS severity		
Mild (1-10)	6 (66.7)	3 (33.3)
Moderate (11-20)	8 (66.7)	4 (33.3)
Sleepiness scale severity (total score)		
Normal (0-5)	6 (100)	0 (0)
Mild (6-10)	8 (66.7)	4 (33.3)
Moderate (11-12)	1 (50)	1 (50)
Severe (13-15)	0 (0)	2 (100)
Very severe (16-24)	0 (0)	0 (0)

## Data Availability

Will be provided upon request.
